# Methotrexate Neurotoxicity Is Related to Epigenetic Modification of the Myelination Process

**DOI:** 10.3390/ijms22136718

**Published:** 2021-06-23

**Authors:** Yu-Chieh Chen, Jiunn-Ming Sheen, Su-Chen Wang, Mei-Hsin Hsu, Chih-Cheng Hsiao, Kow-Aung Chang, Li-Tung Huang

**Affiliations:** 1 Department of Pediatrics, Kaohsiung Chang Gung Memorial Hospital and Chang Gung University College of Medicine, Kaohsiung 833, Taiwan; ray.sheen@gmail.com (J.-M.S.); cuzhengwang@gmail.com (S.-C.W.); a03peggy@cgmh.org.tw (M.-H.H.); chihcheng.hsiao@gmail.com (C.-C.H.); 2 Department of Traditional Medicine, Chang Gung University, Guishan, Taoyuan 333, Taiwan; 3 Department of Anesthesiology, Kaohsiung Chang Gung Memorial Hospital and Chang Gung University College of Medicine, Kaohsiung 833, Taiwan; kowaung@adm.cgmh.org.tw

**Keywords:** methotrexate, neurotrophin, myelination, epigenetic

## Abstract

With the improvement of the survival rate of acute lymphoblastic leukemia (ALL) in children, some children ALL survivors reveal inferior intellectual and cognition outcome. Methotrexate (MTX), while serving as an essential component in ALL treatment, has been reported to be related to various neurologic sequelae. Using combined intrathecal (IT) and intraperitoneal (IP) MTX model, we had demonstrated impaired spatial memory function in developing rats, which can be rescued by melatonin treatment. To elucidate the impact of MTX treatment on the epigenetic modifications of the myelination process, we examined the change of neurotrophin and myelination-related transcriptomes in the present study and found combined IT and IP MTX treatment resulted in altered epigenetic modification on the myelination process, mainly in the hippocampus. Further, melatonin can restore the MTX effect through alterations of the epigenetic pathways.

## 1. Introduction

Cognitive impairment is commonly reported in patients with cancer treated with chemotherapy [1,2]. With improvements in diagnostic and therapeutic strategies, the long-term survival rate of pediatric acute lymphoblastic leukemia (ALL) patients is almost 90% [3,4,5]. However, follow-up studies on childhood leukemia survivors revealed impaired intellectual and cognitive functions [6,7,8], which raised interest in the study of the neurologic impact of chemotherapy medications on pediatric cancer survivors.

Methotrexate (MTX), a folate analog, administered intravenously or by intrathecal (IT) infusion, has been used for more than 60 years for cancer treatment, particularly in ALL [9]. However, clinical studies have shown that MTX chemotherapy is responsible for both functional and morphological changes in the brain, and it can result in serious late neurologic sequelae, notably cognitive impairment [10,11,12]. Most preclinical studies have focused on MTX treatment in adult rodents, but rarely in adolescent rodents. Recently, Wen et al. used models combining intraperitoneal (IP) and IT MTX protocols, which mimicked ALL treatment protocols, and found impaired memory, neuroinflammation, and impaired neurogenesis in developing rats [13]. Recently, we used developing rats treated with IP, IT, or combined IT and IP MTX intended to mimic the treatment model in acute lymphoblastic leukemia in children and found that combined IP and IT MTX treatment caused spatial deficits in developing rats [14]. For better understanding of the underlying mechanism interplay between brain neurotrophin and myelination-related transcriptomes and brain functional impairment, we focus on the myelination process change in response to MTX treatment in this study.

MTX, a commonly used antimetabolite chemotherapeutic agent, is associated with chemotherapy-related cognitive impairment, colloquially known as “chemobrain” or “chemofog” [15,16,17]. Gibson et al. demonstrated that tri-glial dysfunction underlies MTX chemotherapy-related cognitive impairment (CRCI), with direct effects of MTX on microglial activation, subsequently inducing neurotoxic astrocyte reactivity and oligodendroglial lineage dysregulation [18]. Microglial depletion following MTX exposure decreases astrocyte reactivity, normalizes oligodendroglial lineage dynamics and myelination, and rescues cognitive function in a mouse model of juvenile MTX exposure [18]. In our previous study, we found that combined IT and IP MTX treatment resulted in myelin loss; this was evidenced by a decrease in myelin-related protein expression and a decrease in the expression of protein arginine N-methyltransferase 1(PRMT 1), which was considered to be related to the myelination process [14]. Therefore, we presumed that MTX-related cognitive impairment is mediated through myelin loss by directly exerting its effect on the myelination process or through the epigenetic regulation process. Therefore, we tested the effect of MTX treatment on the myelination process in developing rats, mainly focusing on the epigenetic regulation of BDNF and myelination-related transcriptomes in this study.

Recently, there have been increasing studies on melatonin for the elimination of various MTX toxicities, including mucositis, enterocolitis, and renal damage [19,20,21]. However, studies utilizing melatonin to reverse MTX neurotoxicity are lacking. We established an MTX-treated animal model mimicking the pediatric leukemia treatment protocol and tested whether MTX treatment (either through intravenous or intrathecal route) would result in neurologic deficits. Moreover, we tested the therapeutic effects of melatonin on MTX-induced neurotoxicity. Our study revealed that melatonin rescued the MTX-related spatial memory defects, based on the outcomes of the Morris water maze test [14]. Previously, melatonin was reported to rescue MTX neurotoxicity through its role as an oxidative stress scavenger. In this study, we further explored the effect of melatonin treatment on the epigenetic modification of MTX neurotoxicity.

Several epigenetic mechanisms appear to be related to nervous system development. Emerging evidence has revealed that neurogenesis is associated with unique epigenetic features. From the embryonic stage to the adjustment stage, several proteins, including glial fibrillary acidic protein, brain-derived neurotrophic factor (BDNF), and Na-K-Cl-cotransporter 1, participate in neurogenesis. Furthermore, recent studies have shown that impaired epigenetic modification of the gene expression of neurogenetic proteins is related to the pathogenesis of several neurological disorders and the neurotoxicity of various medications. Although epigenetic mechanisms have been implicated in mediating high levels of plasticity during early development, the decreased plasticity and sensitivity that occurs later can also be viewed from an epigenetic perspective. During the onset of critical period plasticity, oligodendrocytes start to express specific myelin structural proteins, including myelin basic protein (MBP), myelin-associated glycoprotein, myelin oligodendrocyte glycoprotein, and myelin-associated oligodendrocyte basic protein. The manipulation of the epigenetic status of oligodendrocytes may also be an effective strategy for modulating plasticity. Sry-related HMg-box (SOX) genes are transcription factors involved in the developmental specification and are classified into nine groups based on sequence similarity and function [22]. Among them, the SOXE group, composed of SOX8, SOX9, and SOX10, is involved in the specification of myelinating glial cells [23]. SOX10 is considered the main SOX transcription factor that regulates oligodendrocyte specification and maturation, as well as epigenetic functions [24]. As growing evidence has revealed the role of SOX10 in the myelination process, especially the aggravation of myelin maturation [25,26], we used SOX10 in this study as a marker to determine the effect of MTX treatment on its expression and epigenetic modification. Besides, in order to evaluate the impact of MTX treatment on the developing brain, we examined the effect of MTX treatment on the epigenetic modifications of myelination-related transcriptomes, including MBP and SOX 10, in a developing rat model.

BDNF is a member of the neurotrophin family of growth factors related to the canonical nerve growth factor. In several brain ischemia/reperfusion models, BDNF is related to neurogenesis and repair [27,28]. Recent studies have demonstrated that BDNF not only potentiates normal central nervous system myelination during development but also enhances recovery after myelin injury [29]. Moreover, Ng et al. found that the serum BDNF concentrations are related to self-perceived cognitive impairment after chemotherapy [30]. Azoulay et al. found that BDNF protein concentrations and gene polymorphisms may be predictors of peripheral neuropathy after chemotherapy [31]. Furthermore, Geraghty et al. found that loss of adaptive myelination contributes to MTX-related cognitive impairment and may be rescued by the manipulation of BDNF expression [32]. In conclusion, the above studies found that MTX decreased cortical BDNF expression, which was restored by microglial depletion. In this study, we tested the degree of BDNF promoter and BDNF mRNA expressions in MTX-treated developing rats. In addition, BDNF promoter methylation status after MTX treatment was examined to evaluate the epigenetic modification of BDNF after MTX treatment, which provides a comprehensive evaluation of the epigenetic modification of the genes involved in the brain myelination process after MTX treatment in the developing brain of developing rats.

Protein arginine methylation, a post-translational modification recognized as important as phosphorylation or ubiquitination, is catalyzed by nine members of the protein arginine methyltransferase family (PRMT1–9) [33]. Most PRMT family members, including PRMT 1, 2, 3, 5, 7, and 8 and coactivator-associated arginine methyltransferase 1 (CARM1, also known as PRMT4), were confirmed to be expressed in the mouse brain. However, compared with other PRMTs, the expression of PRMT1 is observed at earlier stages of development, which implies its importance in stem cell biology and development. In addition, brain cell RNA-seq studies have demonstrated PRMT1 expression in all major central nervous system (CNS) cell types, including neurons, astrocytes, oligodendrocytes, and microglia [33,34]. Recent reports have revealed the significance of PRMT1 in the development of neurons, astrocytes, and oligodendrocytes, although further investigations, particularly of proliferation, differentiation, and development, are needed. Furthermore, several reports have suggested the involvement of PRMT1 in various CNS diseases, including neurodegenerative disorders, brain tumors, and multiple sclerosis [33,34]. These findings suggest that PRMT1 regulates the development and physiological functions of the brain.

Recently, Forster et al. found that altered methylation may potentially act as a mediating mechanism between MTX treatment and subsequent neurotoxicities [35]. Our previous studies have demonstrated that MTX treatment can alter the myelination process, which is presumed to be due to increased oxidative stress. We have found alterations in PRMT1 expression in a previous study, and growing evidence suggests that PRMT1 plays an essential role in post-translational modification through interaction with myelination-related transcriptomes, which implies that an epigenetic mechanism underlies MTX neurotoxicity. However, the PRMT responsible for the methylation of the identified proteins in vivo and the proteins that are real targets of PRMT1 in the brain have not been established. Therefore, in combination with these substrate-enrichment methodologies, we evaluated the role of PRMT1 in the post-translational modification of myelination-related transcriptomes.

## 2. Results

### 2.1. Methotrexate Treatment Resulted in Altered Myelination-Related Protein mRNA Expression

**Figure 1:**
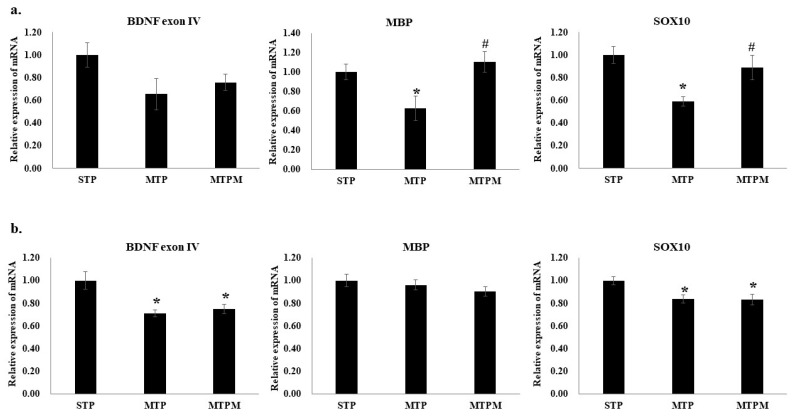
Methotrexate treatment resulted in myelination-related protein mRNA expression alteration; however, melatonin recovery effects were significantly observed in cortex but not hippocampus. (**a**) MTX treatemnt resulted in significant down-regulation of MBP and SOX 10 mRNA expressions in brain cortex while BDNF exon IV was declined but not significatly changed. (**b**) MTX treatemnt resulted in a significant down-regulation of BDNF exon IV and SOX10 mRNA expression while MBP slightly decreased without significance in the hippoccampus (*: *p* < 0.05 vs. STP; #: *p* < 0.05 vs. MTP).

As shown in Figure 1a, the quantifications of BDNF exon IV, MBP, and SOX10 mRNA expression in the cortex via real-time PCR revealed that MTX treatment resulted in significant downregulation of MBP and SOX 10 mRNA expression in brain cortex while BDNF exon IV was declined but not significatly changed (F (2, 15) = 5.666, *p* = 0.015; F (2, 15) = 9.881, *p* = 0.002; F (2, 15) = 2.515, *p* = 0.114, respectively). However, melatonin did not restore the BDNF exon IV mRNA levels, whereas recovery effects were noted in MBP and SOX mRNA expressions. These findings imply that MTX treatment disturbed BDNF, MBP, and SOX10 mRNA expressions, but melatonin rescued only the myelination function and not cognition in the cortex. In the hippocampus, a similar phenomenon was observed. As shown in Figure 1b, MTX treatment resulted in significant downregulation of BDNF exon IV and SOX10 mRNA expressions while MBP slightly decreased without significance (F (2, 18) = 10.401, *p* = 0.001; F (2, 18) = 5.102, *p* = 0.018; F (2, 18) = 1.099, *p* = 0.354, respectively). However, melatonin did not restore the BDNF exon IV, MBP, and SOX10 mRNA levels. These results suggest that MTX treatment disturbed myelination and cognitive functions in the hippocampus. 

### 2.2. Combined Intrathecal and Intraperitoneal Methotrexate Treatment Resulted in an Altered CpG Methylation Status of the BDNF Exon IV Promoter in Both the Brain Cortex and Hippocampus

**Figure 2:**
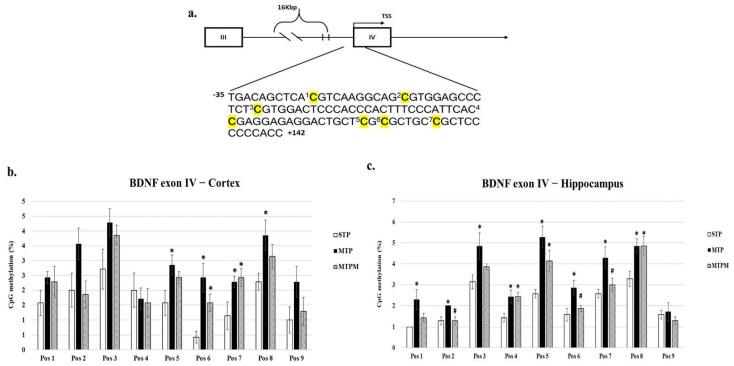
MTX treatment stimulated the alteration of CpG methylation status in BDNF exon IV promoter while melatonin attenuated the alteration resulted from MTX. (**a**) Scheme of BDNF exon IV promoter region for pyrosequencing. CpG positions are highlighted in yellow background. (**b**) Methylation status in different CpG sites of BDNF exon IV promoter in brain cortex. (**c**) Methylation status in different CpG sites of BDNF exon IV promoter in hippocampus (* *p* < 0.05 vs. STP; # *p* < 0.05 vs. MTP). “C” highlights in bold with yellow background indicate variable CpG positions.

As shown in Figure 2, pyrosequencing examination of the cortex revealed increased methylation of CpG at positions 5, 6, 7, and 8 (Pos5: F (2, 18) = 3.780, *p* = 0.043; Pos6: F (2, 18) = 8.377, *p* = 0.003; Pos7: F (2, 18) = 4.469, *p* = 0.027; Pos8: F (2, 18) = 3.689, *p* = 0.045, Figure 2b). In the hippocampus, increased methylation of the CpG at positions 1–8 was noted (Pos1: F (2, 18) = 4.846, *p* = 0.021; Pos2: F (2, 18) = 7.500, *p* = 0.004; Pos3: F (2, 18) = 4.139, *p* = 0.033; Pos4: F (2, 18) = 5.880, *p* = 0.011; Pos5: F (2, 18) = 6.091, *p* = 0.010; Pos6: F (2, 18) = 6.091, *p* = 0.010; Pos7: F (2, 18) = 5.850, *p* = 0.011; Pos8: F (2, 18) = 5.418, *p* = 0.014; respectively, Figure 2c). However, the degree of methylation status alteration in response to melatonin treatment varied in the brain cortex and hippocampus (Figure 2b,c).

### 2.3. Combined Intrathecal and Intraperitoneal Methotrexate Treatment Resulted in Altered CpG Methylation Status of the SOX10 and MBP Promoter in Both Brain Cortex and Hippocampus

**Figure 3:**
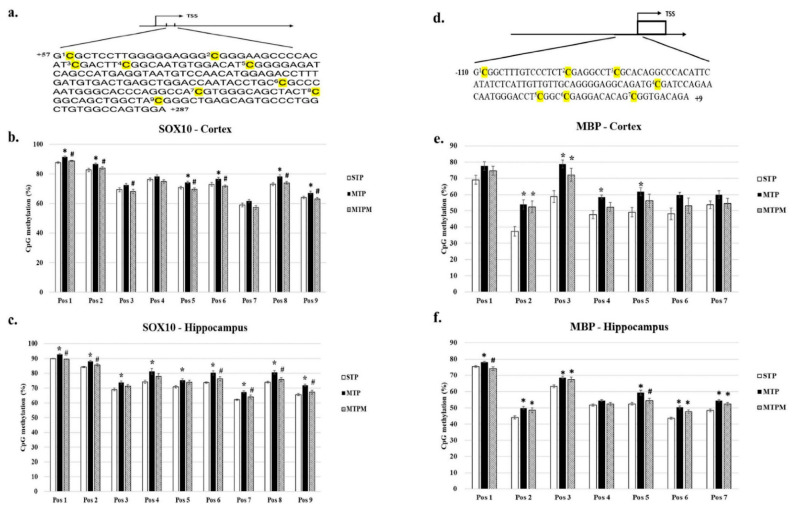
MTX treatment stimulated the alteration of CpG methylation status in myelination-related genes promoter, while melatonin attenuated the alteration resulted from MTX. (**a**) Scheme of SOX10 promoter region for pyrosequencing. CpG positions are highlighted in yellow background. (**b**) Methylation status in different CpG sites of SOX10 promoter in brain cortex. (**c**) Methylation status in different CpG sites of SOX10 promoter in hippocampus. (**d**) Scheme of MBP promoter region for pyrosequencing. CpG positions are highlighted in yellow background. (**e**) Methylation status in different CpG sites of MBP promoter in brain cortex. (**f**) Methylation status in different CpG sites of MBP promoter in hippocampus. “C” highlights in bold with yellow background indicate variable CpG positions. (*: *p* < 0.05 vs. STP; #: *p* < 0.05 vs. MTP).

As shown in Figure 3, the CpG methylation status of the SOX10 promoter in the brain cortex examined by pyrosequencing revealed an increased methylation of CpG at positions 1, 2, 5, 6, 8, and 9 (Pos 1: F (2, 18) = 14.546, *p* = 0.000; Pos 2: F (2, 18) = 5.425, *p* = 0.014; Pos 5: F (2, 18) = 8.231, *p* = 0.003; Pos 6: F (2, 18) = 6.394, *p* = 0.008; Pos 8: F (2, 18) =7.952, *p* = 0.003; Pos 9: F (2, 18) = 5.891, *p* = 0.011) after MTX treatment (Figure 3b). In the hippocampus, the upregulation of CpG methylation was significantly observed at positions 1–9 (Pos 1: F (2, 18) = 61.174, *p* = 0.000; Pos 2: F (2, 18) = 9.265, *p* = 0.002; Pos 3: F (2, 18) = 4.651, *p* = 0.024; Pos 4: F (2, 18) = 4.615, *p* = 0.024; Pos 5: F (2, 18) = 3.933, *p* = 0.038; Pos 6: F (2, 18) = 6.831, *p* = 0.006; Pos 7: F (2, 18) = 6.844, *p* = 0.006; Pos 8: F (2, 18) = 8.759, *p* = 0.002; Pos 9: F (2, 18) = 10.233, *p* = 0.001) in the MTP groups (Figure 3c). For MBP, the upregulation of the CpG methylation status at positions 2, 3, 4, and 5 (Pos 2: F (2, 18) = 8.237, *p* = 0.003; Pos 3: F (2, 18) = 8.437, *p* = 0.003; Pos 4: F (2, 18) = 5.996, *p* = 0.010; Pos 5: F (2, 18) = 3.848, *p* = 0.041) in the brain cortex and the upregulation of the CpG methylation status at positions 1, 2, 3, 5, 6, and 7 (Pos 1: F (2, 18) = 6.786, *p* = 0.006; Pos 2: F (2, 18) = 6.454, *p* = 0.008; Pos 3: F (2, 18) = 7.152, *p* = 0.005; Pos 5: F (2, 18) = 7.651, *p* = 0.004; Pos 6: F (2, 18) = 13.078, *p* = 0.000; Pos 7: F (2, 18) = 14.299, *p* = 0.000) in the hippocampus were noted (Figure 3e,f). Similar to its effect on BDNF methylation status, melatonin treatment resulted in a varied response of methylation status changes in the SOX10 and MBP promoters. Taken together, these findings suggest that the hippocampus is more susceptible to MTX treatment than the brain cortex in relation to changes in methylation status.

### 2.4. Combined Intrathecal and Intraperitoneal Methotrexate Treatment Resulted in Epigenetic Modification of BDNF Exon IV Promoter, Myelin Basic Protein (MBP) and SOX 10 Promoter

**Figure 4:**
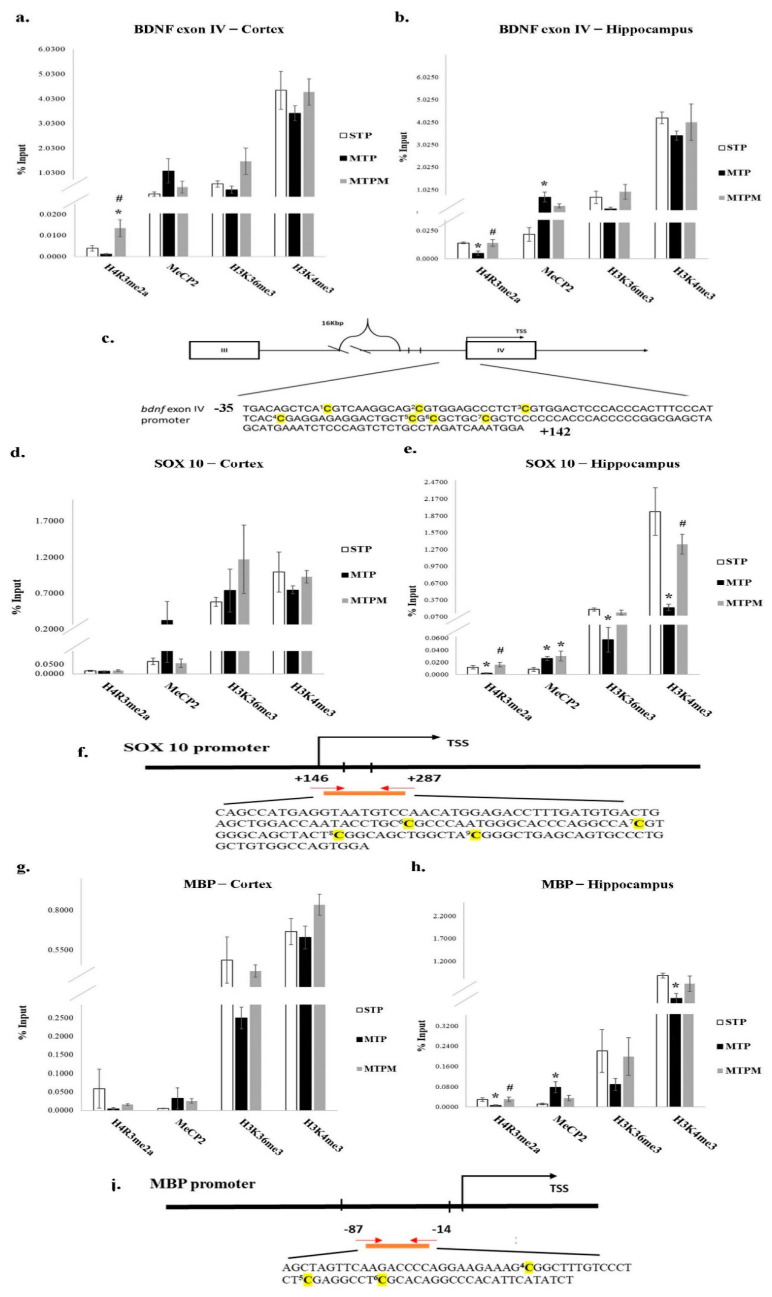
MTX treatment altered epigenetic modification of BDNF exon IV and myelination-related gene promoter, however, melatonin prevented the alteration induced by MTX. (**a**) ChIP exam for BDNF exon IV promoter revealed decrease in the level of H4R3me2a and increase in the level of MeCP2 inbrain cortex. (**b**) ChIP exam for BDNF exon IV revealed significant decrease in the level of H4R3me2a and increase in the level of MeCP2 in the hippocampus and in the brain cortex, respectively. (**c**) BDNF IV promoter sequences for ChIP assay. (**d**) ChIP exam for SOX 10 promoter found less significant effect of MTX in brain cortex. (**e**) ChIP exam for SOX10 promoter found significant change in the levels of H4R3me2a, MeCP2, H3K36me3, and H3K4me3 in the hippocampus. (**f**) SOX10 promoter sequences for ChIP assay. (**g**) ChIP exam for MBP promoter decrease in the level of H4R3me2a and increase in the level of MeCP2 in brain cortex; (**h**) ChIP exam for MBP promoter revealed significant alteration of the expression levels in hippocampus. (**i**) MBP promoter sequences for ChIP assay. “C” highlights in bold with yellow background indicate variable CpG positions. (*: *p* < 0.05 vs. STP; #: *p* < 0.05 vs. MTP).

As shown in Figure 4, ChIP was carried out with anti-histone H4 dimethyl arg3 asymmetric (H4R3me2a), anti-methyl-CpG binding protein 2 (MeCP2), anti-histone H3 trimethyl lysine 36 (H3K36me3), and anti-histone H3 trimethyl lysine 4 (H3K4me3). In brain cortex, while statistically insignificant, decrease in the level of H4R3me2a and increase in the level of MeCP2 were noted after MTX treatment (H4R3me2a: F (2, 9) = 6.561, *p* = 0.017; H3K4me3: F (2, 9) = 0.828, *p* = 0.468; MeCP2: F (2, 9) = 2.271, *p* = 0.159; H3K36me3: F (2, 9) = 3.366, *p* = 0.081) (Figure 4a). In hippocampus, significant decrease in the level of H4R3me2a and increase in the level of MeCP2 were noted after MTX treatment (H4R3me2a: F (2, 9) = 6.298, *p* = 0.019; MeCP2: F (2, 9) = 6.452, *p* = 0.018; H3K36me3: F (2, 9) = 2.332, *p* = 0.153; H3K4me3: F (2, 9) = 0.661, *p* = 0.540, Figure 4b). Interestingly, melatonin treatment successfully rescued the MTX effect on the alteration of the level of H4R3me2a in both brain cortex and hippocampus (F (2, 9) = 2.271, *p* = 0.0159; H4R3me2a: F (2, 9) = 6.298, respectively, Figure 4a,b). 

As for myelination related proteins, significant alteration of the levels of H4R3me2a, MeCP2, H3K36me3, and H3K4me3 of SOX10 promoter was noted after MTX treatment in the hippocampus (H4R3me2a: F (2, 9) = 7.334, *p* = 0.013; MeCP2: F (2, 9) = 4.808 *p* = 0.038; H3K36me3 F (2, 9) = 4.644, *p* = 0.041, H3K4me3 F (2, 9) = 10.601, *p* = 0.004) while the effect of MTX on brain cortex was less significant (H4R3me2a: F (2, 9) = 0.447, *p* = 0.653; H3K4me3: F (2, 9) = 0.567, *p* = 0.586; MeCP2: F (2, 9) = 0.984, *p* = 0.411; H3K36me3: F (2, 9) = 0.886, *p* = 0.445, Figure 4d,e). In addition, study on the levels of H4R3me2a, MeCP2, H3K36me3, and H3K4me3 of MBP promoter region revealed significant alteration of the expression levels in hippocampus (H4R3me2a: F (2, 9) = 4.331, *p* = 0.048; H3K4me3: F (2, 9) = 4.486, *p* = 0.044; MeCP2: F (2, 9) = 5.515, *p* = 0.027; H3K36me3: F (2, 9) = 1.126, *p* = 0.366) compared with brain cortex (H4R3me2a: F (2, 9) = 0.895, *p* = 0.442; H3K4me3: F (2, 9) = 2.209, *p* = 0.166; MeCP2: F (2, 9) = 0.738, *p* = 0.505; H3K36me3: F (2, 9) = 1.847, *p* = 0.213). (Figure 4g,h). Following melatonin treatment, H4R3me2a level seemed to recover both in SOX 10 and MBP promoter region in hippocampus (SOX10 in cotrex: F (2, 9) = 0.447, *p* = 0.653, hippocampus: F (2, 9) = 7.334, *p* = 0.013; MBP in cortex: F (2, 9) = 0.895, *p* = 0.442, hippocampus: F (2, 9) = 4.331, *p* = 0.048, Figure 4e,h). The above results suggested that MTX treatment modulated MBP and SOX10 expression through alteration of H4R3me2a and MeCP2-binding affinity of promoter regions in hippocampus. Besides, H4R3me2a, a genes activator which catalyzes asymmetric dimethylation of H4R3 by protein arginine methyltransferase 1 (PRMT1), was found to moderately decrease in the promoter region 2 after MTX treatment, which implied that the epigenetic modifications after MTX treaetment may be induced by the alterations of the methyltransferase activities. Further, melatonin seemed to exert its effect on the epigenetic modification through H4R3me2a in the myelination proteins, mostly in the hippocampus.

### 2.5. PRMT1 Is Involved in the Post-Translational Modification of BDNF and Myelination-Related Transcriptomes in MTX-Treated Developing Rats

**Figure 5:**
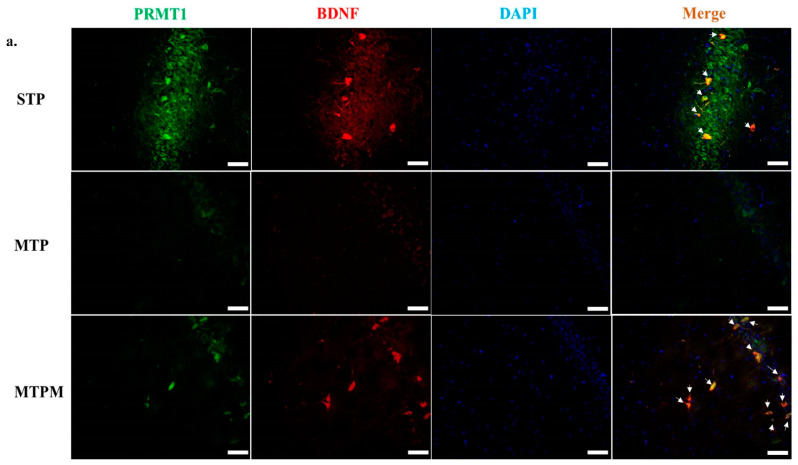

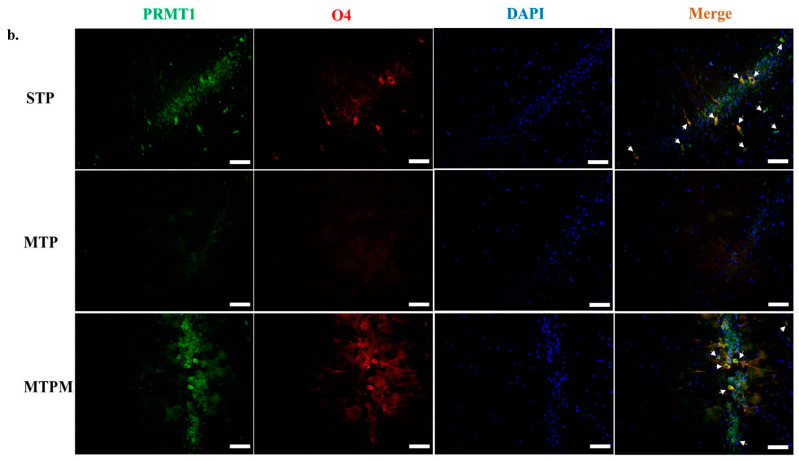
Interactions of PRMT1 with BDNF and oligodendrocyte progenitor cells were disturbed by MTX treatment. In contrast, melatonin prevented the disturbances of MTX. (**a**) Immunofluorescence staining of PRMT1 and BDNF in the cornu ammonis 1 (CA1) area of hippocampus CA1 area revealed colocalization of PRMT1 and BDNF. MTX treatment resulted in a decrease in staining of both PRMT1 and BDNF, and melatonin could reverse the effect. Hippocampus CA1 double-stained PRMT1 (Green), BDNF (Red), DAPI (Blue) indicated nuclei. (**b**) Immunofluorescence staining of the early stage oligodendrocyte progenitor cells (O4 cells) revealed decrease in PRMT1 expression MTX treatment and the effect could be rescued by melatonin treatment. Hippocampus CA1 double-stained PRMT1 (Green) and oligodendrocyte marker O4 (Red), DAPI (Blue) indicated nuclei. The arrow indicated co-localization of PRMT1/BDNF or PRMT1/O4 in CA1 area. Each bar indicated 50 μm.

As shown in Figure 5, immunofluorescence staining of PRMT1 and BDNF in the cornu ammonis 1 (CA1) area of the hippocampal CA1 area revealed colocalization of PRMT1 and BDNF in the sham groups. After MTX treatment, decreased staining of both PRMT1 and BDNF was noted, which recovered after melatonin treatment and could be colocalized (Figure 5a). Since the present study focused mostly on the developing brain, we further checked PRMT1 expression by immunofluorescence-staining changes in early stage oligodendrocyte progenitor cells, which are O4 cells, after MTX treatment. We found decreased PRMT1 expression in O4 cells after MTX treatment, which could be rescued by melatonin treatment (Figure 5b). The above results indicated that PRMT1 was involved in the myelination of the developing brain and suggested that melatonin rescued the MTX effect by serving a role other than oxidant scavenging.

**Figure 6:**
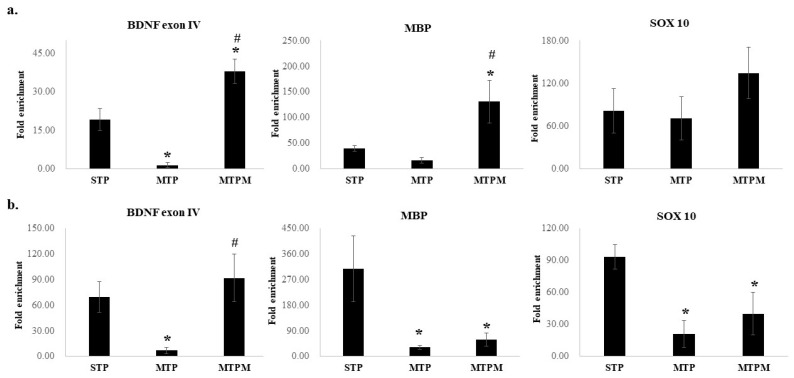
RPMT 1 regulated BDNF exon IV and myelination-related transcriptomes were repressed by MTX treatment while melatonin restored it. (**a**) RIP exam found significant decrease of BDNF exon IV and decrease of MBP and SOX 10 promoter in brain cortex. (**b**) RIP exam found significant decrease of BDNF exon IV, MBP, and SOX 10 in hippocampus. (* *p* < 0.05 vs. STP; # *p* < 0.05 vs. MTP).

To further investigate the role of the post-translational modification of the myelination-related transcriptomes by PRMT1, we used the RIP method to study the interaction between PRMT1 and the RNA molecules of BDNF exon IV, MBP, and SOX10 (Figure 6). A significant decrease in the BDNF exon IV was found in both the brain cortex and hippocampus (cortex: F (2, 9) = 23.466, *p* = 0.000; hippocampus: F (2, 9) = 5.182, *p* = 0.032), which could be successfully rescued by melatonin (Figure 6). The expression levels of MBP and SOX10 in the hippocampus were significantly decreased (MBP: F (2, 9) = 4.947, *p* = 0.036; SOX10: F (2, 9) = 6.125, *p* = 0.021, Figure 6b). In summary, the above findings suggest that PRMT1 plays an essential role in the post-translational modification of BDNF, MBP, and SOX10, while the hippocampus was more strongly affected by MTX treatment.

## 3. Discussion

Since we have demonstrated that combined IT and IP MTX treatment resulted in spatial memory deficits in developing rats, we conducted this study to uncover the underlying mechanisms, and the findings are summarized below. First, combined intrathecal and intraperitoneal methotrexate treatment resulted in both functional and epigenetic changes in the developing brain. Second, the hippocampus is more susceptible to MTX treatment, mostly due to epigenetic modifications. Third, the interaction of PRMT1 with the translational process (mRNA expression) by RIP in our study provides evidence that PRMT1 may be involved in the process of myelination dysregulation in MTX neurotoxicity. Fourth, our study provides a treatment option for MTX neuropathy using medications targeting the epigenetic pathway, possibly through H3R4me2a. Finally, melatonin rescues MTX neurotoxicity not only through its antioxidant effects, but also by modulating the epigenetic process.

Nowadays, improvement of cancer survival has been reported following integration of multiple treatment strategies. However, CRCI is still a hard task for cancer survivors and attracts much attention. Previously, it has been suggested that CRCI is a consequence of cytokine release following chemotherapy [36,37]. Recently, more and more studies claimed that not only the direct effect of chemotherapy or inflammation response induced by chemotherapy on the nervous system, but the impact of the chemotherapy on neuron plasticity and repair leads to the development of CRCI. Koh et al. has found that the role of cancer exosomes and their ability to interact with the nervous system is essential in modulating neurological processes such as neuronal functioning and stress response, which might also contribute to CRCI [38,39]. Similar to Koh’s report, our work emphasizes the alteration of neuronal plasticity through the change in the myelination process contributes to CRCI. In addition, it deserves to note that despite growing studies work on the mechanism of CRCI, there was only one study to discuss the epigenetic impact on the development of CRCI [40]. To date, there are limited studies to discuss the impact of epigenetic programming and CRCI [40,41]. Wang et al. stated that chemobrain was related to the epigenetic reprogramming of the cancer patients and the cytokine response to the chemotherapy [41]. Unlike Wang’s work, our study emphasizes the epigenetic changes on the myelination process following MTX treatment and is considered to be the first study to directly examine the brain myelination-related transcriptomes change after chemotherapy. Moreover, to investigate the CRCI from the view of exosome dynamics in the future would make the work more comprehensive.

Chemobrain by MTX was previously considered to be due to the dysregulation of oxidative stress. Gibson et al. examined the postmortem specimen after MTX chemotherapy and found persistent tri-glial dysfunction underlies MTX chemotherapy-related cognitive impairment [18]. Similar to our study, Gibson et al. used a mouse model mimicking leukemia treatment protocol to examine the effect of MTX exposure on juvenile rats and found microglial depletion, decreased astrocyte reactivity, altered oligodendroglial lineage dynamics [18]. Furthermore, Geraghty et al. demonstrated that loss of adaptive myelination contributes to MTX chemotherapy-related cognitive impairment through the evidence that microglial-dependent reduction of BDNF expression and loss of activity-regulated myelination [32]. Consistent with our findings, these results indicate that the dysregulation of the myelination process underlies the mechanism of chemobrain following MTX.

To date, only one study conducted by Forster et al. has investigated the role of MTX on DNA methylation in the pathogenetic process of MTX neurotoxicity [42]. However, Forster et al. studied the impact of MTX on epigenetic modification using normal neural cell lines and emphasized that depletion of S-adenosyl methionine had an impact on myelination [42]. In this study, we found that MTX treatment resulted in the alteration of epigenetic modifications of the neurotrophin gene and myelination-related proteins gene expression, while the concept that neurotoxicity following MTX treatment may be a result of the dysregulation of the epigenetic process in neurotrophin genes has rarely been reported. Moreover, we examined the neurotrophin and myelination-related proteins gene expression in brain cortex and hippocampus rather than cell lines. Therefore, we suggested that MTX neurotoxicity was not only a consequence of an imbalance in oxidative stress, but also due to a change in the epigenetic regulation of neurotrophin and myelination-related transcriptomes. 

The impact of MTX treatment on the nervous system most commonly manifests as spatial memory impairment, which was considered to be due to its effect on the hippocampus. In this study, epigenetic changes after MTX treatment were mostly found in the hippocampus, with minimal effects on the brain cortex. This finding seems to be correlated with the results of brain morphology and functional study after MTX treatment. According to the literature, distribution of MTX after either intravenous or intraventricular injection is uneven [43,44,45]. Westerhout et al. demonstrated that the spatial distribution in CNS of unbound MTX after intraventricular administration was significantly related to the distance from the injection site. In addition, the balance between brain extracellular fluid status and cerebrospinal fluid (CSF) determined also the spatial distribution of MTX [45]. Furthermore, the diseased neuron and the healthy neuron had difference in susceptibility of MTX, thus the impact of MTX on the diseased neuron and the healthy neuron is different. To this end, we conclude that the hippocampus is more susceptible to MTX treatment in both functional and neurogenesis aspects, which might be explained by the higher MTX concentration in hippocampus than brain cortex. However, since we used healthy animals in the study, we suggested that the finding should be interpretated cautiously because the MTX concentration in the real patients is different from the health subjects.

Using the ChIP method, we found a significant decrease in the concentration of H4R3me2a and an increase in the concentration of MeCP2 in the hippocampus in the BDNF exon IV promoter region, which was successfully rescued by melatonin treatment by altering the concentrations of H4R3me2a in the brain cortex and hippocampus. In addition, significant changes in the concentrations of H4R3me2a, MeCP2, H3K36me3, and H3K4me3 in the SOX10 promoter region were noted after MTX treatment in the hippocampus, while the effect of MTX on the brain cortex was less significant. Further, studies on the concentrations of H4R3me2a, MeCP2, H3K36me3, and H3K4me3 in the MBP promoter region revealed significant alterations in the expression levels in the hippocampus compared with the brain cortex. Following melatonin treatment, the H4R3me2a concentrations seemed to be recovered in both the SOX 10 and MBP promoter regions in the hippocampus. Taken together, we found that MTX treatment modulated MBP and SOX10 expressions through the alteration of the H4R3me2a and MeCP2-binding affinities of the promoter regions in the hippocampus, and melatonin exerted its effect on epigenetic modification through H4R3me2a in the myelination proteins mostly in the hippocampus. To the best our knowledge, this is the first study to illustrate that melatonin may work through epigenetic modifications to rescue the effect of MTX on the CNS. 

In our previous study, we found that PRMT1 expression was altered by combined IT and IP MTX treatment. Moreover, increasing evidence suggests that PRMT1 plays an essential role in post-translational modification through interaction with myelination-related transcriptomes. Therefore, we used substrate-enrichment methodologies to evaluate the role of PRMT1 in the post-translational modification of myelination-related transcriptomes. Using the RIP method, we found a significant decrease in BDNF exon IV in both the brain cortex and hippocampus, which can be successfully rescued by melatonin. In addition, there was a significant decrease in the expression levels of MBP and SOX 10 in the hippocampus. These findings suggest that PRMT1 plays an essential role in the posttranslational modification of BDNF, MBP, and SOX10, and the finding that the hippocampus was more deeply affected by MTX treatment was consistent with the formal studies. 

Despite we have tried great effort to elucidate the mechanism of MTX neurotoxicity through epigenetic reprogramming on myelination process in the developing brain, several unsolved questions remain. First, since we have found that PRMT1 is involved in the myelination process in response to MTX treatment, the key substrates of PRMT1 regulation of neurogenesis and repair processes remain unclear. The antibody-based PRMT1 substrate-enrichment strategy utilized may not have captured all substrates because PRMT1 also acts on substrates without typical arginine-glycine-rich motifs. Second, the interrelationship between PRMT1 and other PRMTs, in terms of the functional regulation of the brain after MTX treatment, was not explored in the present study. For a more comprehensive intervention of the role of PRMTs in the epigenetic modification of the genes of neurotrophin and myelination-related transcriptomes, further studies on other PRMTs are required. Third, since PRMT1 protein concentrations have been shown to gradually increase during brain development and become maintained in adult brains [34], PRMT1 has stage-dependent functions which may further change in a disease-related fashion. Fourth, differential spatial distribution of MTX in brain warrants further study to explain the different extent of involvement in epigenetic programming in cortex and hippocampus. Addressing these remaining questions, notably utilizing exosomes as means to explore different body compartments will lead to a further understanding of mechanism of MTX neurotoxicity.

## 4. Material and Methods

### 4.1. Subjects

This experiment was performed per the Guidelines for Animal Experiments of Chang Gung Memorial Hospital and Chang Gung University. The experiments were approved by the Institutional Animal Care and Use Committee, Taiwan (approval number: 2018032204; approval date: 2 May 2018). The day of delivery was defined as day 0. Male Sprague-Dawley rats (PND 17 ± 1) weighing 50 g were used. Attempts were made to minimize the number of animals used. All animals were housed in a room maintained at 24 °C a 12-h light/dark cycle and had free access to standard chow. 

According to Forsythe et al. higher incidence rates of childhood acute lymphoblastic leukemia was found in boys compared to girls [42]. Besides, since we used melatonin as the therapeutic agent, its anti-estrogen effect was ever reported [46,47]. Furthermore, this study was an extended research from our previous study [14], which used Morris water maze (MWM) to determine the neurobehavior changes and thus built up a male rats MWM database. Therefore, we choose male rats as our target subjects in this study. 

### 4.2. Experimental Procedures

All surgical procedures were performed under anesthesia with Zoletil50 (25 mg/kg) and xylazine (23 mg/kg), as previously described [48]. Each group of rats received an intrathecal injection via a transcutaneous cisternal magna puncture. Briefly, the rats were anesthetized and positioned in the lateral decubitus position. A 27-gauge needle was inserted into the cisterna magna. The correct position was verified by the outflow of CSF, and the polyethylene catheters were inserted through a small incision in the atlantooccipital membrane and passed 1.5 cm caudally to the level of the lumbar enlargement of the catheters [49]. To confirm the correct placement of the catheters, 10 μL of 0.9% saline was flushed into them the day after surgery. 

As we have already demonstrated that combined MTX-intrathecal and intraperitoneal treatment may result in significant spatial memory changes along with a marked decrease in brain-related protein expression and decreased myelination, we focused on the combined MTX-intrathecal and intraperitoneal groups in the present study. The dosages of MTX were selected based on our preliminary experiments and previous reports [13,14,50]. 

There were three experimental groups in this project (N = 10–14, each).

1. Sham (STP): rats that underwent catheter implantation surgery received an equal volume of normal saline with MTX treatment via intrathecal injection (IT) and intraperitoneal injection (IP). 

2. MTP: rats that underwent catheter implantation surgery received 0.5 mg/kg of methotrexate diluted with 0.9% normal saline in 5–10 µL as the final volume via intrathecal injection once per week for two weeks. The rats received 100 mg/kg methotrexate via i.p. injection 24 h after each dose of IT injection. 

3. MTPM: rats that received intraperitoneal melatonin treatment of 100 mg/kg of melatonin 1 h before methotrexate treatment (IT + IP).


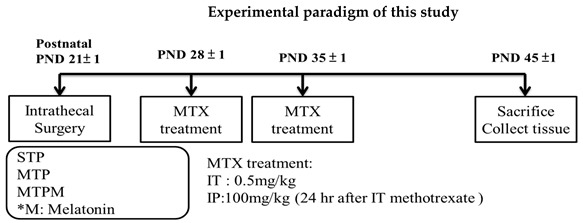


### 4.3. Brain Tissue Collection

The brain tissue was removed and homogenized immediately after the rats were sacrificed at PND 45. We examine two regions that are involved in spatial performance in rats: the prefrontal cortex and hippocampus [51,52]. The prefrontal cortex and hippocampus are removed immediately after rats are sacrificed, and tissues are homogenized. Before collection, all brain tissues were perfused with 0.9% iced saline. The hippocampus is primarily involved in cognition and spatial performance, thus we examined exclusively the hippocampus in the immunofluorescence staining [52,53]. 

### 4.4. DNA Bisulfite Modification and Pyrosequencing

According to a previous study, DNA from the prefrontal cortex or hippocampus tissue was used in the study, and genomic DNA extraction was performed using the tissue genomic DNA extraction mini kit (Favorgen Biotech Corp, Taipei, Taiwan) [54]. In brief, 500 ng of genomic DNA was used for bisulfite modification (EZ DNA Methylation-Gold Kit, Zymo Research, Irvine, CA, USA). DNA methylation level quantitation analysis was conducted using pyrosequencing, as previously described. The gene promoter of interest was amplified from bisulfite-converted DNA by PCR. To isolate the single-stranded amplicon, the anti-sense primers used in PCR reactions were specially designed with a biotin moiety at the 5′ terminus. A total PCR reaction with a volume of 25 μL was performed, which involved 2 μL of the bisulfite-modified DNA template, PyroMark PCR Master Mix (Qiagen, Hilden, Germany), oral load concentrate, 25 mM MgCl_2_, and 10 μM each of gene-specific forward and reverse primers. Thermocycling was conducted using the following general parameters: 95 °C for 15 min, followed by 50 cycles at 95 °C for 30 s, 58–60 °C for 30 s, 72 °C for 1 min, and final extension at 72 °C for 10 min. Following amplification, 10–20 μL of PCR product was mixed with streptavidin-conjugated sepharose beads (GE Healthcare, Boston, MA, USA) in a binding buffer (Qiagen, Germantown, MD, USA) and diluted to 80 μL total volume with ddH2O. The beads were subsequently collected using a vacuum preparation workstation and sequencing primer and heated to 82 °C for 2 min. The sequencing primers, shown in Table 1, were annealed to the biotinylated DNA strand as the reaction mixture was cooled to room temperature. Pyrosequencing was performed using a PyroMark Q24 system (Qiagen, Hilden, Germany). The peak heights of the CpG sites were calculated and converted into percentages of C/C+T representing methylation status using PyroMark Q24 Advanced software (version: 3.0.0; Build: 21).

### 4.5. Chromatin Immunoprecipitation (ChIP) Assay for BDNF and Myelination-Related Transcriptomes Modification

Chromatin immunoprecipitation was performed as previously described using the Magna ChIP™ A/G Chromatin Immunoprecipitation Kit (Millipore, Billerica, MA, USA) according to the manufacturer’s instructions [36,55,56,57]. Sheared chromatin (90 μL) from the prefrontal cortex or hippocampus was used for immunoprecipitation and incubated at 4 °C overnight with primary antibodies (anti-H4R3me2a, anti-H3K4me3, antiH3K36me3, and anti-MeCP2; Abcam, Cambridge, UK) or normal rabbit IgG (negative control, supplied in the kit). Meanwhile, 5 μL of sheared chromatin was saved as input for normalization. Follow-up purified gDNA from each antibody was used at the BDNF exon IV, MBP, and SOX10 promoter region via real-time PCR (Table 2). The percentage input was calculated using the following formula: ΔCt [normalized ChIP] = (Ct [ChIP] − (Ct [Input] − Log2 (Input dilution factor), where dilution factor = 5/90 = 18.5. Finally, input%  =  100/2^ΔCt [normalized ChIP]^, with this value delegating the enrichment of epigenetic modifications in specific regions. 

### 4.6. RNA-Protein Immunoprecipitation (RIP) Assay

RIP was performed using an EZ-Magna RIP Kit (Millipore, Billerica, MA, USA) according to the manufacturer’s instructions. Cells were lysed using a complete RNA lysis buffer with protease inhibitor and RNase. Total of 100 μL of cell lysate was incubated with RIP buffer containing magnetic beads conjugated with antibodies of PRMT1 (1:80X) or with negative control (IgG), and 10 μL of cell lysate was used as the input in the following procedure. Wash buffer was added to all RIP reactions and vortexed gently, and the wash steps were repeated until the magnetic beads were washed five times. Thus, purified RNAs from different immunoprecipitated antibodies were transcribed into cDNA and analyzed for BDNF exon IV, MBP, and SOX10 mRNA expression levels via real-time PCR. The values were calculated as fold enrichment = 2^(ΔCt(IP) − ΔCt(mock))^.

### 4.7. Quantitative Real-Time PCR Analysis

PCR analysis was performed as previously reported [48]. The parameters examined included BDNF exon IV, MBP, and SOX10 (Table 3). 

### 4.8. Immunofluorescence Staining

Cryosections of brain tissue were cut in a facial orientation of 16 μm thickness and used in the following procedures, as previously described (14). Anti-PRMT1 antibody (Rabbit; Abcam, Cambridge, UK), anti-BDNF (Mouse; Proteintech, IL, USA), and anti-oligodendrocyte marker 4 (O4, Mouse; STEMCELL Technologies Inc., Vancouver, BC, Canada) antibody were diluted in ratios of 1:100, 1:100, and 1:50 in phosphate buffer saline (PBS, pH 7.4) and incubated overnight at 4 °C. Specific protein distributions in the hippocampus were detected using Alexa Fluor-594 at a 1:100 ratio (Thermo Fisher, Waltham, MA, USA), conjugated with anti-mouse antibody and Alexa Fluor-488 at a 1:500 ratio (Thermo Fisher, Waltham, MA, USA), and conjugated with anti-rabbit antibody. Finally, the slices were mounted and imaged using a microscope (Olympus, Tokyo, Japan).

### 4.9. Statistical Analysis

PCR, pyrosequencing, and ChIP were analyzed using one-way ANOVA with an LSD post-hoc test. All analyses were performed using SPSS on a PC-compatible computer. The values are expressed as mean ± SEM, and significance was set at *p* < 0.05 for all tests.

## 5. Conclusions

In conclusion, the study indicated that an epigenetic process underlies the pathogenesis of MTX neurotoxicity, mainly in the neurogenesis and myelination processes. Furthermore, this is the first study to address the interaction between PRMT1 and neurotrophin and myelination-related transcriptomes in post-translational regulation. 

## Figures and Tables

**Table 1:** Primer set for pyrosequencing.

Gene	Forward (5′-3′)	Reverse (5′-3′) Biotinylated	Sequencing Primer
*BDNF* exon IV	AGGTAGAGGAGGTATTATATGATAGT AGGTAGAGGAGGTATTATATGATAGT	ACTATATATTTCCCCTTCTCTTCAATT AACTCTTACTATATATTTCCCCTTCT	AGGAGGTATTATATGATAGTT ATTTATAGAGGAGAGGATTGT
MBP	GAGATAGTTAGTTTAAGATTTTAGGAAGAA	ACCCTACCAATTATTCTTTAAATCTACT	GTTAGTTTAAGATTTTAGGAAGAAA
*SOX10*	GTTAGGTAAGGTAGATTTTAAAAGGGATG	TAACCACAACCAAAACACTACTCAA	GTAGATTTTAAAAGGGATGG ATGTGATTGAGTTGGAT

**Table 2:** Primer set for ChIP assay.

	Forward 5′-3′	Reverse 5′-3′
BDNF exon IV promoter	TGACAGCTCACGTCAAGGCA	ATCTAGGCAGAGACTGGGAGAT
MBP promoter	AGCTAGTTCAAGACCCCAGG	AGATATGAATGTGGGCCTGTGC
SOX10 promoter	CAGCCATGAGGTAATGTCCA	GTAGCCAGCTGCCGAGTAG

**Table 3:** Pirmer design for real-time PCR.

	Forward 5′-3′	Reverse 5′-3′
BDNF exon IV	ATTCACCGAGGAGAGGACTG	AGTCTTTGGTGGCCGATATG
MBP	CTCTGGCAAGG CTCACACAC	TCTGCTGAGGGACAGGCCTCTC
SOX10	TACAAGTACCAACCTCGGCG	CATGGGGGAGCCTTCTTCTG
